# Antimicrobial resistance among bacteria isolated from urinary tract infections in females in Namibia, 2016–2017

**DOI:** 10.1186/s13756-022-01066-2

**Published:** 2022-02-12

**Authors:** Erastus H. Haindongo, Binta Funtua, Boni Singu, Marius Hedimbi, Francis Kalemeera, Jana Hamman, Olli Vainio, Antti J. Hakanen, Jaana Vuopio

**Affiliations:** 1 School of Medicine, Faculty of Health Sciences, University of Namibia, Windhoek, Namibia; 2 Institute of Biomedicine, University of Turku, Turku, Finland; 3 School of Pharmacy, Faculty of Health Sciences, University of Namibia, Windhoek, Namibia; 4 Namibia Institute of Pathology, Windhoek, Namibia; 5 Faculty of Medicine, University of Turku, Turku, Finland; 6 Department of Clinical Microbiology, Laboratory Division, Turku University Hospital, Turku, Finland

**Keywords:** Antimicrobial resistance, Urinary tract infections, Female, *Escherichia coli*, Uropathogens, Namibia

## Abstract

**Background:**

The emergence of antimicrobial resistance (AMR) among bacterial pathogens demands a local understanding of the epidemiological situation. This information is needed both for clinical treatment decision-making purposes as well as for the revision of current care guidelines. Clinical AMR data from Namibia is sparse, whilst urinary tract infections remain not only widespread but they disproportionally affect females. This paper aims to describe the national antimicrobial resistance situation of major bacterial uropathogens in females within the 14 Namibian regions.

**Method:**

Retrospective countrywide information on clinical urine cultures performed in females in Namibia in 2016–2017 was obtained from the national public health laboratory, Namibia Institute of Pathology (NIP). The data set included both microbiological findings as well as antimicrobial susceptibility test (AST) results. The AST was done as per the Clinical and Laboratory Standards Institute (CLSI) guidelines. Resistance to 3rd generation cephalosporins was indicative of Extended Spectrum-ß-lactamase (ESBL) production. Data analysis was done with WHONET using expert interpretation rules.

**Results:**

In total, 22,259 urinary cultures were performed, of which 13,673 (61.4%) were culture positive. Gram-negative bacterial species accounted for 72.6% of the findings. The most common pathogens identified were *Escherichia coli*, *Klebsiella pneumoniae* and *Proteus mirabilis*. Most of these were from young females, with a median age ranging from 28 to 32 years for the various pathogens. Resistance to ampicillin was 77.7% *in E. coli* and 84.9% in *K. pneumoniae*. In *E. coli,* resistance to 1^st^ line empiric therapy antibiotic, nitrofurantoin, was below 13%, except for one region that showed 59.2% resistance*.* Resistance to third generation cephalosporin (3GC) was used as a proxy for ESBL production. By year 2017, 3GC resistance was 22%, 31.4% and 8.3% for *E. coli, K. pneumoniae* and *P. mirabilis,* respectively*.*

**Conclusion:**

We report high resistance to ampicillin, quinolones and sulfamethoxazole-trimethoprim amongst *E. coli*. Resistance rates to third-generation cephalosporins was also concerningly high at 22%. Resistance to carbapenems was low. However, superiority of nitrofurantoin was found, which provides rational support for the usefulness of nitrofurantoin as an empiric therapy regimen for the treatment of urinary tract infections in this setting.

**Supplementary Information:**

The online version contains supplementary material available at 10.1186/s13756-022-01066-2.

## Background

Antimicrobial resistance (AMR) is an increasing global health issue that generally threatens public health [1, 2]. Urinary tract infections (UTI) are common and disproportionally affect women [3–5], with at least 150 million infections annually [6]. Resistance to empirical antimicrobial therapeutics has reportedly increased [7–10], particularly amongst gram-negative bacteria [11].

Continuous surveillance is especially needed in low and middle-income countries due to their high burden of infections. Surveillance carries the benefit of informing therapy guidelines [12, 13]. It is thus important that both surveillance and reporting are standardized [14] to allow for comparability across settings.

Namibia’s surveillance capacity for resistant pathogens needs strengthening [15]. Furthermore, reports on antimicrobial resistance from Namibia are also limited [16, 17] and the resistance situation of many pathogenic isolates from different specimen types have not been described.

This study reports on the antimicrobial resistance situation of selected pathogenic micro-organisms causing UTI in females using retrospective laboratory AST surveillance data gathered from the Namibian public health laboratory service, the Namibia Institute of Pathology (NIP).

### Aim/objectives

The aim was to describe the national antimicrobial resistance situation of common uropathogens in Namibian females.

## Materials and methods

### Study setting: urine specimen collection

Specimens were obtained from both in- and out-patients presenting with clinical symptoms indicative of a possible UTI and were obtained before the start of antimicrobial therapy.

### Bacterial isolation and identification

The culture positivity cut-off was ≥ 10^3^ CFU/ml, following 18–48 h of incubating an inoculated media plate at 37 °C aerobically. Cultures with 4 or more organisms were classified as contaminated specimens. Isolate identifications were done using Analytical Profile Index (API-bioMériuex, Marcy l’Etoile, France) 10S or 20E GNB and VITEK®2 XL (API-bioMériuex, Marcy l’Etoile, France) GN cards.

### Antimicrobial susceptibility testing (AST) procedure

AST was performed in accordance with the CLSI M100 ED26 & ED27 guidelines. At the NIP Central Laboratory in Windhoek, AST was performed with the commercial VITEK®2 XL system with AST N255 cards and at the peripheral laboratories with the Kirby-Bauer disk diffusion method.

The AST disks were: amoxicillin-clavulanic acid (20/10 μg), ampicillin (10 μg), cefepime (30 μg), cefotaxime (30 μg), ceftazidime (30 μg), ceftriaxone (30 μg), cefuroxime (30 μg), cephalothin (30 µg), gentamicin (10 μg), imipenem (10 μg), nalidixic acid (30 μg), nitrofurantoin (300 μg), ofloxacin (5 μg), piperacillin-tazobactam (100/10 μg), sulfamethoxazole/trimethoprim (1.25/23.75 μg). Quality control included weekly testing of susceptible *Escherichia coli* strain ATCC 25,922.

Resistance to any of the 3rd generation cephalosporins (3GC) was used to infer ESBL production. The outcomes (Resistant, Intermediate and Susceptibility i.e. RIS category, Zone of Inhibition measurements and MIC’s) from the network of laboratories were captured in MEDITECH–a centralized laboratory information system.

### Data acquisition and analysis

A datafile containing microbiological information (including AST results) of isolates from female urine cultures between January 2016 and December 2017 was exported from MEDITECH. This anonymized datafile contained countrywide information on the age of the patient, hospital location, specimen type, specimen collection date, species/name of the micro-organism found and antimicrobial susceptibility test results based on the RIS classification for every isolate. The RIS determinations were made with WHONET 2019 (VITEK MICs only) using the CLSI M100 ED29:2019 interpretative breakpoints.

## Results

### Specimens and culture overview

The total number of microbiological cultures performed on female urine specimens between 2016 and 2017 was 22,259 with a culture positivity rate of 62.6% and 60.4% in the respective 2 years (Table 1).

**Table 1:** Overview of urine cultures performed (N = 22,259) at NIP by year

	2016	2017	Total
Urine specimens	n (%)	n (%)	n (%)
Culture negative	3922 (37.4)	4664 (39.6)	8586 (38.6)
Culture positive	6555 (62.6)	7118 (60.4)	13,673 (61.4)
Total	10,477 (100)	11,782 (100)	22,259 (100)

**Fig. 1:**
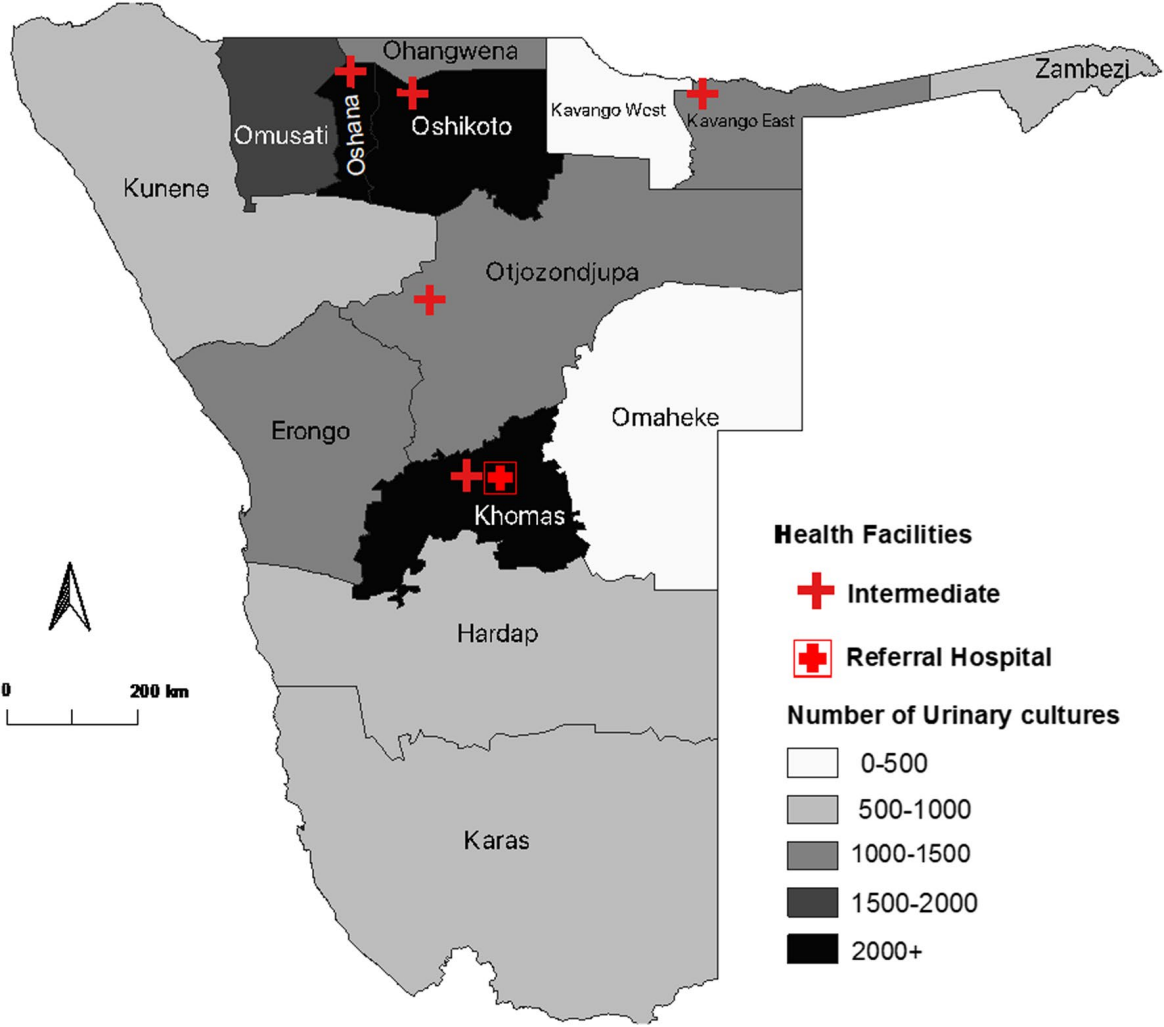
Number of urine cultures performed in 2016–2017 by region and location of intermediate and referral hospitals in Namibia. Primary care (i.e. clinics) and district hospitals not shown

The numbers of urine specimens and cultures were highest in Khomas, Oshana, Oshikoto, and Omusati. These regions recorded over 2,000 cultures in the 2 years combined (Fig. 1).

The majority of isolates were *E. coli* (40.7%)*, K. pneumoniae* (6.3%) *and P. mirabilis* (5.4%) by species. Proportionally, all other gram-negatives and gram-positives were 5% and less of the total isolates. The aforementioned three species will thus be referred to as common offending pathogens in this paper. For these offending pathogens, the proportions were very comparable during the two years (Table 2).

**Table 2:** Proportions of microbiological findings in 2016–2017

	2016	2017	Total
Microbiological findings	n (%)	n (%)	n (%)
Gram negatives			
*Escherichia coli*	2659 (40.6)	2909 (40.9)	5568 (40.7)
*Klebsiella pneumoniae*	390 (6.0)	472 (6.6)	862 (6.3)
*Proteus mirabilis*	359 (5.5)	385 (5.4)	744 (5.4)
other gram negatives	1436 (22)	1321 (18.6)	2757 (20.2)
Gram positives	1030 (15.7)	1077 (15.1)	2107 (15.4)
Mixed bacterial growth	278 (4.2)	499 (7.0)	777 (5.7)
Fungi	398 (6.1)	449 (6.3)	847 (6.2)
Clostridium *spp*	0	4*	4*
Mycobacteria	3*	0	3*
Helminthes	2*	2*	4*
Total	6555 (100)	7118 (100)	13,673 (100)

^*^Less than 1%

The distribution of the common uropathogens across the regions generally followed the same trend as that of the frequency of urine specimens collected (see Fig. 1). The majority of bacterial organisms were isolated from the capital Khomas region, i.e. more than 20% of the isolates. The Kavango East, Omusati, Oshana and Oshikoto regions which are predominantly north of the capital region, ranked in the top five of the regions with significantly high isolate counts after the Khomas region.

### Patient demographic information

The age distribution data shows that the median age for UTI patients was between 28 and 32 years (Additional file 1: Supplementary 1), except in the Kavango East region where the median age was 110. The inter-quartile age range of patients with an *E. coli* isolate was 23–47 and 23–45 years in 2016 and 2017, respectively. Furthermore, the three common pathogens were generally isolated from young to middle-aged adult females in the age range of 18–52 years. Among the 0–4 years age group, *Klebsiella pneumoniae* was proportionally the most common isolate (i.e. 16.9% and 13.1% in 2016 and 2017, respectively) whilst *Proteus mirabilis* was more common in the older age groups (i.e. > 60 years).

### Antimicrobial resistance by antimicrobial group

#### *E. coli* ß-lactams ± inhibitor

*E. coli* resistance was highest for ampicillin, being 75.8% in 2016 and 77.7% in 2017. Amoxicillin-clavulanic acid resistance was 12.3% and 19.3% in 2016 and 2017, respectively. From 2016–2017, cefuroxime resistance varied from 18.0% to 22.4% and of 3GCs, ceftriaxone resistance was of the same level*.* ESBL confirmatory tests were performed in one region only and thus the exact ESBL rates could not be given. Resistance to carbapenems was almost non-existent (< 1%) (Table 3).

**Table 3:** Antimicrobial resistance of *E. coli* in 2016–2017 in Namibia, 2016–2017

	2016 (n = 2659)			2017 (n = 2909)		
Antimicrobials	No. of Resistant isolates	No. of isolates tested	%Resistant	No. of Resistant isolates	No. of isolates tested	%Resistant
Ampicillin	410	541	75.8	580	746	77.7
Amoxicillin-Clavulanic acid	153	1245	12.3	299	1551	19.3
Piperacillin/Tazobactam	4	112	3.6	7	186	3.8
Cephalothin	481	1467	32.8	564	1345	41.9
Cefuroxime	213	1186	18.0	268	1195	22.4
Ceftriaxone	83	460	18.0	73	332	22.0
Ceftazidime	15	67	22.4	8	76	10.5
Imipenem	4	470	0.9	4	713	0.6
Nalidixic acid	651	1700	38.3	671	1744	38.5
Ciprofloxacin	180	935	19.3	160	982	16.3
Ofloxacin	265	1699	15.6	290	1790	16.2
Gentamicin	309	1734	17.8	340	1819	18.7
Nitrofurantoin	163	1730	9.4	224	1803	12.4
SXT	1257	1550	81.1	1031	1336	77.2

SXT: Sulfamethoxazole/Trimethoprim; **%**: Percentage

#### Quinolone/fluoroquinolone

Quinolone resistance, i.e. nalidixic acid resistance, was 38.3% and 38.5% in 2016 and 2017, respectively. Whereas ciprofloxacin and ofloxacin resistance was in the range of 15.6–19.3% during the two consecutive years (Table 3).

#### Nitrofurantoin

The first-line empiric antibiotic nitrofurantoin generally showed low resistance rates, ranging from 9.4 to 12.4% in 2016 and 2017, respectively. However, resistance to nitrofurantoin was exceptional in the Zambezi region, with a resistance rate of 59.2% (45/76) and 48.9% (43/88) in 2016 and 2017, respectively.

#### Sulfamethoxazole-trimethoprim

Resistance to sulfamethoxazole-trimethoprim was 81.1% and 77.2% in 2016 and 2017, respectively.

#### *Klebsiella pneumoniae* and *Proteus mirabilis*

*Klebsiella pneumoniae* resistance to ampicillin was 84.9% in both years. Amoxicillin-clavulanic acid was 13.8% and 12.7% in the two years. Cefuroxime resistance was 29.0% and 28.6%. *K. pneumoniae* exhibited resistance to ceftriaxone at 32.7% and 31.4%. Only one isolate out of 171 isolates tested was found to be resistant to imipenem. (Table 4).

**Table 4:** Antimicrobial resistance of *Klebsiella pneumoniae* in Namibia, 2016–2017

	2016 (N = 390)			2017 (N = 472)		
Antimicrobials	No. of Resistant isolates	No. of isolates tested	%Resistant	No. of Resistant isolates	No. of isolates tested	%Resistant
Ampicillin	73	86	84.9	101	119	84.9
Amoxicillin/Clavulanic acid	23	167	13.8	32	251	12.7
Piperacillin/Tazobactam	0	15	0	0	29	0
Cephalothin	81	192	42.2	89	215	41.4
Cefuroxime	40	138	29	42	147	28.6
Ceftriaxone	18	55	32.7	11	35	31.4
Ceftazidime	2	5	40	1	7	14.3
Imipenem	0	50	0	1	121	0.8
Nalidixic acid	35	210	16.7	58	267	21.7
Ciprofloxacin	18	96	18.8	17	114	14.9
Ofloxacin	20	215	9.3	29	287	10.1
Gentamicin	69	217	31.8	70	277	25.3
Nitrofurantoin	50	213	23.5	65	280	23.2
SXT	125	194	64.4	127	209	60.8

SXT: sulfamethoxazole/trimethoprim; **%**: percentage

**Table 5:** Antimicrobial resistance of *Proteus mirabilis* in Namibia, 2016–2017

	2016 (N = 359)			2017 (N = 385)		
Antimicrobials	No. of Resistant isolates	No. of isolates tested	%Resistant	No. of Resistant isolates	No. of isolates tested	%Resistant
Ampicillin	34	88	38.6	84	167	50.3
Amoxicillin/clavulanic acid	13	195	6.7	37	259	14.3
Piperacillin/tazobactam	2	15	13.3	0	25	0
Cephalothin	27	207	13	46	190	24.2
Cefuroxime	20	175	11.4	16	168	9.5
Ceftriaxone	6	60	10	3	36	8.3
Ceftazidime	1	6	16.7	1	4	25
Ciprofloxacin	16	136	11.8	6	133	4.5
Gentamicin	37	260	14.2	36	273	13.2
Imipenem	3	56	5.4	5	90	5.6
Nalidixic acid	49	256	19.1	56	268	20.9
Nitrofurantoin	182	257	70.8	191	268	71.3
Ofloxacin	21	253	8.3	18	273	6.6
SXT	134	229	58.5	110	188	58.5

SXT: Sulfamethoxazole/Trimethoprim; %: Percentage

Among *P. mirabilis,* 3GCs resistance ranged from 8.3 to 11.4% whereas resistance to imipenem was 5.4% and 5.6 in the two consecutive years. Nitrofurantoin resistance was 70.8% and 71.3% in 2016 and 2017 whereas sulfamethoxazole-trimethoprim was 58.5% in both years (Table 5).

## Discussion

To our knowledge this is the first report on antimicrobial resistance rates among urinary pathogens in Namibian women. *E. coli* was found to be the main pathogen with high resistance rates to ampicillin, fluoroquinolones and sulfamethoxazole-trimethoprim. Twenty-two percent of the isolates were resistant to third-generation cephalosporins which is concerning. However, resistance to carbapenems was low.

The empirical drug, nitrofurantoin has demonstrated superiority in our setting.

In our nationwide collection of urine culture specimens, the culture positivity rate of 61.4% found is somewhat comparable to the 51.2% positivity found by Rizvi et al. among pregnant women in India [18] or reported from Western countries by Hooton et al. [19]. As the culture practices and guidance of either the laboratories or the clinicians have not changed in Namibia during the recent years, we expect the figures to represent the current situation. There is no universal agreement on the cut-off value that represents a positive urine culture. Across different settings cut-off values are set between 10^2^ to 10^5^ CFU/ml and may potentially overestimate or underestimate culture positivity rates [20, 21]. In Namibia a cut-off value of ≥ 10^3^ CFU/ml is used. Other factors which have reported to influence culture positivity rates include individual and population level treatment-seeking behaviour [22] and diagnostic methods with variable sensitivity or better Positive Predictive Values (PPV) than traditional culture [21, 23, 24].

The Khomas, Oshana, Omusati and Oshikoto regions had the highest number of positive urinary culture findings. Intermediate-high level (i.e. referral hospital) care facilities are primarily located in the aforementioned regions, but we do not have further information on the distribution of out- and in-patients in our study material.

Gram-negative organisms accounted for at least 70% of the isolates cultured, with the most common pathogens being *E. coli* and* K. pneumoniae,* which is in concordance with the aetiological findings reported elsewhere [5, 22]. The Global Antimicrobial Surveillance System (GLASS) recommends reporting resistance for priority pathogens, *E. coli* and *K. pneumoniae* from urine specimens [14].

We report that young and middle-aged adults are mostly affected by urinary tract infections. This is consistent with the risk age for UTI’s that has been reported in other settings elsewhere [25, 26]. Age shifts to the elderly were noticeable in regions that are primarily rural, particularly the Kavango East Region. The Kavango East region is an economically disadvantaged region. In Namibia, only the elderly are exempted from in-and-outpatient healthcare service fees, which may reflect to health service usage.

High AMR rates (~ 80%) were reported in *E.coli* against ampicillin and sulfamethoxazole/trimethoprim. This is very similar to that of neighboring South Africa [27]. These antimicrobials should thus only be used in clinical treatment when supported by AST results to minimize the risk of treatment failure. Similar to Kenya, these antibiotics are used in Namibia for respiratory illness and pneumocystis prophylaxis in HIV infected individuals, which may fuel the situation respectively [28].

Resistance rates in the range of 12.3–22.4% were reported to several antibiotics, namely amoxicillin-clavulanic acid, cefuroxime and gentamicin (Tables 4, 5). Resistance to nalidixic acid is in the range of 38%, whilst ofloxacin resistance remained at ~ 16%. Nitrofurantoin resistance rates were the lowest being in the range of 9.4–12.4%.

The Namibia Standard Treatment Guidelines (NSTG) recommends the use of nitrofurantoin and nalidixic acid for cystitis in adults and children, respectively. Intravenous gentamicin and cefuroxime are recommended for upper UTI or complicated presentations [29]. Nitrofurantoin has demonstrated high susceptibility against *E. coli,* whilst nalidixic acid, gentamicin and cefuroxime showed variable but worryingly high resistance rates.

ESBL producers were inferred from 3GCs resistance, and for *E.coli* the ESBL rates represented 18.0–22.0% in our study. This is within the global ESBL-resistance ranges of 15–75% and 28–68% against cefotaxime reported in 2020 and 2021, respectively [30, 31]. Across Europe, *E. coli—*3GC resistance ranged from 6.2 to 30.8% among bacteremic isolates in 2019 [32]. Muriuki *et.al.* reports a similar finding among *E. coli* uropathogens in Kenya between 2015 and 2018 [33]. Nonetheless an ESBL prevalence of 25% significantly creates therapeutic problems with ß-lactams and quinolones [34].

In *K. pneumoniae,* likewise to *E. coli*, relatively high resistance levels were observed*.* Especially, 3GC resistance was noteworthily high (%R: 32.7%). The global cefotaxime resistance ranges stood at 32–62% and 28–62% in 2020 and 2021, respectively [30, 31]. In European countries this rate has varied between 4.3 and 75.7% in 2019 [32]. Fortunately, only one carbapenemase producing isolate among those tested was found.

To our slight surprise, *Proteus mirabilis* stood as the third most common UTI pathogens with worrying resistance against key antimicrobials. This organism may threaten treatment success with the empiric nitrofurantoin in rural regions due to the high resistance (> 70%).

We recognize various systematic and methodological limitations which should be considered when interpreting our findings. The Namibian healthcare system lacks a unique patient identifier and the laboratory relies on specimen and requisition numbers for identification and traceability. Patients with multiple specimen submissions could not be identified in order to apply the often recommended first isolate rule to the analysis.

Our records did not document the setting of infection acquisition (out- or in-patient/ward/unit), patient characteristics and other epidemiological information (i.e. pregnancy and other predisposing conditions). We were thus not able to perform sub-group analysis by setting or patient characteristics, which would have allowed more in depth analysis for antimicrobial therapy guidance purposes.

Due to operational and financial constraints, laboratories across the country do not have similar resources or equipment. This may have affected the AST practices performed (including the decision on when and for which antibiotics AST is performed) even though, in general, the same standard operation procedures (SOP) are instructed to be followed in all NIP laboratories. For example, of 5568 *E. coli* isolates cultured, only approx. 63% were tested against the first line empirical drug, nitrofurantoin. Also, the semi-automated commercial diagnostic systems such as VITEK are only found at the central main laboratory in Windhoek, while the other laboratories use solely disk diffusion method for AST. Consequently, due to possible lack of uniformity in microbiological practices, resistance rates reported may be overestimated or underestimated across sites. There is thus a need for the strengthening of standardization of testing to further increase the reliability of the results.

## Conclusion

This is the first paper to describe the antimicrobial resistance situation of urine isolates in Namibia. Moderate to high resistance levels to several empirical UTI antibiotics were observed, and one fifth of *E. coli* isolates showed third-generation cephalosporin resistance. However, superiority of nitrofurantoin was found, which provides rational support for the usefulness of nitrofurantoin as an empiric therapy regimen for the treatment of urinary tract infections in Namibia.

## Supplementary Information

**Additional file 1.** Patient age distribution by cultured pathogen and year.

## Data Availability

Datasets used and/or analysed during the current study are available from the corresponding author on reasonable request.

## Abbreviations

%R: Percentage resistance
3GC: Third-generation cephalosporin
95–95%: Confidence Interval
AMC: Amoxicillin-clavulanic acid
AMR: Antimicrobial Resistance
AST: Antimicrobial Susceptibility Test
CLSI: Clinical and Laboratory Standards Institute
ESBL: Extended Spectrum-ß-lactamases
IQR: Interquartile range
NA: Missing
SXT: Sulphamethoxazole/trimethoprim
